# Clinical course, mutations and its functional characteristics of infantile-onset Pompe disease in Thailand

**DOI:** 10.1186/s12881-019-0878-8

**Published:** 2019-09-11

**Authors:** Lukana Ngiwsara, Duangrurdee Wattanasirichaigoon, Thipwimol Tim-Aroon, Kitiwan Rojnueangnit, Saisuda Noojaroen, Arthaporn Khongkraparn, Phannee Sawangareetrakul, James R. Ketudat-Cairns, Ratana Charoenwattanasatien, Voraratt Champattanachai, Chulaluck Kuptanon, Suthipong Pangkanon, Jisnuson Svasti

**Affiliations:** 1 Laboratory of Biochemistry, Chulabhorn Research Institute, Bangkok, Thailand; 2 Division of Medical Genetics, Department of Pediatrics, Faculty of Medicine Ramathibodi Hospital, Mahidol University, Bangkok, Thailand; 3 Pediatrics Department, Faculty of Medicine, Thammasat University, Pathumthani, Thailand; 4 School of Chemistry, Institute of Science, Suranaree University of Technology, Nakhon Ratchasima, Thailand; 5 Current address: Synchrotron Light Research Institute, Nakhon Ratchasima, Thailand; 6 Queen Sirikit National Institute of Child Health, Bangkok, Thailand

**Keywords:** Lysosomal strorage disorder, Pompe disease, Glycogen storage disease type II, Acid alpha glucosidase, *GAA*

## Abstract

**Background:**

Pompe disease is a lysosomal storage disorder caused by the deficiency of acid alpha-glucosidase (EC. 3.2.1.20) due to mutations in human *GAA* gene. The objective of the present study was to examine clinical and molecular characteristics of infantile-onset Pompe disease (IOPD) in Thailand.

**Methods:**

Twelve patients with infantile-onset Pompe disease (IOPD) including 10 Thai and two other Asian ethnicities were enrolled. To examine the molecular characteristics of Pompe patients, *GAA* gene was analyzed by PCR amplification and direct Sanger-sequencing of 20 exons coding region. The novel mutations were transiently transfected in COS-7 cells for functional verification. The severity of the mutation was rated by study of the GAA enzyme activity detected in transfected cells and culture media, as well as the quantity and quality of the proper sized GAA protein demonstrated by western blot analysis. The GAA three dimensional structures were visualized by PyMol software tool.

**Results:**

All patients had hypertrophic cardiomyopathy, generalized muscle weakness, and undetectable or < 1% of GAA normal activity. Three patients received enzyme replacement therapy with variable outcome depending on the age of the start of enzyme replacement therapy (ERT). Seventeen pathogenic mutations including four novel variants: c.876C > G (p.Tyr292X), c.1226insG (p.Asp409GlyfsX95), c.1538G > A (p.Asp513Gly), c.1895 T > G (p.Leu632Arg), and a previously reported rare allele of unknown significance: c.781G > A (p.Ala261Thr) were identified. The rating system ranked p.Tyr292X, p. Asp513Gly and p. Leu632Arg as class “B” and p. Ala261Thr as class “D” or “E”. These novel mutations were located in the N-terminal beta-sheet domain and the catalytic domain.

**Conclusions:**

The present study provides useful information on the mutations of *GAA* gene in the underrepresented population of Asia which are more diverse than previously described and showing the hotspots in exons 14 and 5, accounting for 62% of mutant alleles. Almost all mutations identified are in class A/B. These data can benefit rapid molecular diagnosis of IOPD and severity rating of the mutations can serve as a partial substitute for cross reactive immunological material (CRIM) study.

## Background

Pompe disease or glycogen storage disease type II (Pompe disease, GSDII, acid maltase deficiency; OMIM; 232,300) is an autosomal recessive lysosomal storage disorder. The disease results from deficiency of alpha glucosidase (GAA) (EC. 3.2.1.20) which catalyzes the hydrolysis of glycogen to glucose, leading to the accumulation of glycogen in lysosomes of skeletal and cardiac muscles, liver and other tissues, with a variety of clinical symptoms [1–3]. The clinical spectrum is classified into infantile-onset Pompe disease (IOPD) and late-onset Pompe disease (LOPD). IOPD is characterized by onset before 12 months with predominant hypertrophic cardiomyopathy, generalized muscle weakness, hepatomegaly, feeding difficulties, and respiratory compromise. Without treatment by enzyme replacement therapy (ERT), patients with IOPD usually succumb to early death by the age of 2 years due to cardiopulmonary failure. LOPD is characterized by slowly progressive proximal muscle weakness and respiratory insufficiency, either in individuals with onset before 12 months without cardiomyopathy, or in individuals with onset after 12 months. LOPD often leads to the necessity of a wheel chair and respiratory support as well as a higher mortality rate compared with healthy individuals [4]. The incidence of Pompe disease has been reported to be about ~ 1 in 40,000 live births around the world [5].

The *GAA* gene is located on chromosome 17q25.2–25.3, spanning approximately 20 kb and containing 20 exons. The *GAA* cDNA has a coding sequence of 2859 bps in length and encodes a polypeptide of 952 amino acids with a calculated molecular weight of 105 kDa [6, 7]. Normally, GAA protein is synthesized as a 110-kDa precursor containing *N*-linked carbohydrates, and is then modified with mannose 6-phosphate groups [8]. After transport through the Golgi complex and targeting to the endosome/lysosome, the GAA precursor is proteolytically processed and undergoes N-glycan processing, resulting in 95 kDa intermediate forms and finally a 76 and 70 kDa mature protein [9].

Over 550 *GAA* variants are listed in The Pompe Disease Mutation Database (http://www.pompecenter.nl updated on May 2016) [10]. Nearly 70% of the variants listed are marked as pathogenic, while another 10% of the variants have unknown clinical significance. Genotype-phenotype correlation has been observed in Pompe disease to some extent. Infants who have IOPD with no cross reactive immunological material or CRIM-negative tend to have null mutations, whereas missense and splicing variants may lead to IOPD or LOPD [11, 12]. Some specific mutations are correlated with a specific subtype of Pompe disease, such as c.525delT and c.2482_2646del (exon 18 deletion) being associated with IOPD in the Dutch population [13], p.Asp645Glu associated with IOPD of Taiwanese descendants [14], and c.336-13 T > G associated with LOPD [15, 16]. CRIM status is crucial in prediction of the long-term clinical outcome of ERT and the development of antibodies against the enzyme infused. To prevent such antibody production which may reduce response to treatment, CRIM-negative patients require immunomodulation before the initiation of ERT [3]. Therefore, analysis of CRIM status is recommended for every IOPD patients [3]. Since performing CRIM study in cultured fibroblasts is not practical, a format for severity rating which has been proposed by Kroos et al. [17], is recommended to determine if the pathogenic mutation results in complete absence of GAA (CRIM-negative).

Little information is available on *GAA* mutations from the Southeast Asian population. Herein, we performed clinical analysis of patients with IOPD, characterization of *GAA* variants and functional study of the novel mutations identified, and rating its severity.

## Methods

### Patients

Patients with IOPD based on clinical and/or enzymatic diagnosis during 2000–2018 in the participating hospitals were enrolled in the study. Medical records were reviewed. For genetic analysis, written informed consent was obtained from all participants and the parents of the minor participants, after ethics approval was obtained from the Ramathibodi Hospital Institutional Review Board (protocol ID 06–55-46).

### Leukocyte preparation and enzyme assay

Leukocytes were isolated from the whole blood samples as previously published [18]. The assay protocol to measure the acid α-glucosidase activity was performed as previously described [19].

### Mutation analysis

Genomic DNA was extracted from the whole blood using QIAamp DNA Mini kit (QIAGEN GmbH). Primers were designed for all 20 exons and flanking exon-intron boundaries of the *GAA* gene, using primer3 Input version 0.4.0 (http://bioinfo.ut.ee/primer3-0.4.0) [20]. PCR of all exons including the noncoding exon (exon 1) was performed and subsequently subjected to capillary Sanger sequencing in both directions, at Macrogen, Inc. (Seoul, S. Korea). Multiple protein sequence alignment of vertebrate species: *Homo sapiens* (GenBank accession no. ABI53718.1), *Pan troglodytes* (GenBank accession no. JAA43384.1), *Bos Taurus* (GenBank accession no. DAA33199.1), *Mus musculus* (GenBank accession no. AAH10210.1) and *Rattus norvegicus* (GenBank accession no. AAH61753.1)*,* was performed by using Clustal Omega solfware (https://www.ebi.ac.uk/Tools/msa/clustalo). In silico analysis tools were used to predict the pathogenicity of newly identified missense mutations.

### Functional analysis by expression in COS-7 cells and western blot analysis

#### Construction of vectors for GAA mutants and expression in COS-7 cells

To verify the altered function of three novel nonsense/missense mutations, p.Tyr292X, p.Asp513Gly and p.Leu632Arg and the p.Ala261Thr variants (ClinVar accession number VCV000456438.1), site-directed mutagenesis (QuikChange Lightning Site-Directed Mutagenesis Kit; Agilent Technology Inc., Santa Clara, CA, USA) was used to introduce those mutations in wild type *GAA* sequence in pcDNA3.1/CT-GFP-TOPO plasmid (Invitrogen, Carlsbad, CA, USA). The full-length *GAA* cDNA of a normal healthy control was PCR-amplified with the primers cDNA-GAA-F: 5′-GCGGTTGATGTCTCAGAGC-3′ and cDNA-GAA-R: 5′-CTCCAGGTGTCACATGCAAC-3′), and cloned into the pcDNA3.1/CT-GFP-TOPO. Primers used for mutagenesis are shown in Table 1. The methods for transient transfection of cos-7 cells were as described previously [18]. These cells were used for western blot analysis and measurement of GAA activity. The experiments were performed in triplicate.

**Table 1:** Oligonucleotide primers used for site-directed mutagenesis in generation of GAA constructs

Primer	Sequence
A261T-F	5’-ACAGGCCTCACCGAGCACCTC-3’
A261T-R	5’-GAGGTGCTCGGTGAGGCCTGT-3’
Y292X-F	5’-GCGAACCTCTAGGGGTCTCACCC-3’
Y292X-R	5’-GGGTGAGACCCCTAGAGGTTCGC-3’
D513G-F	5’-CAGGTGCCCTTCGGCGGCATGTGGATTG-3’
D513G-R	5’**-**CAATCCACATGCCGCCGAAGGGACCTG-3’
L632R-F	5’-GCCAGAAATCCGGCAGTTTAACC-3’
L632R-R	5’-GGTTAAACTGCCGGAATTTCTGGC-3’

#### Western blot analysis

We used the methodology previously described by Ngiwsara et al., [18]. In the present study, the primary antibody used was a rabbit monoclonal anti-human GAA antibody (dilution 1:1000; #ab137068; Abcam, Cambridge, MA, USA) and the secondary antibody was polyclonal swine anti-rabbit immunoglobulin conjugated with horseradish peroxidase (dilution 1:2000; #P0217; Dako; Agilent Technologies, Inc., Santa Clara, CA, USA).

### Severity rating of the mutations

The severity of novel mutations identified and the p.Ala261Thr variant was determined by a rating system as previously described [15]. According to this system, two types of assay were performed to analyze the impact of sequence variations as follow: 1) GAA enzyme activity detected in transiently transfected COS-7 cells (C%) and in culture media (M%), as expressed in cells transfected with mutant compared to wild type GAA; 2) The combination of outcome data in term of quantity and quality of apparent molecular mass of the secreted precursor 110 kDa protein in medium (M110), intracellular precursor 110 kDa (C110), intermediate 95 kDa (C95), and mature 76 kDa (C76) GAA protein, as visualized by SDS-PAGE and immunoblotting. These are given by rating into two digits. The first digit represents quantity data as follow: 1-not detectable, 2-barely detectable, 3-clearly present, but clearly less than normal, 4-normal, and 5-clearly more than normal. The second digit representing quality data is as the following: 1-too little present to judge, 2-apparent molecular mass too low, 3-apparent molecular mass too high, and 4-apparent molecular mass normal. In brief, the severity of mutation was classified into six classes as follows: A-very severe, B-potentially less severe, C-less severe, D-potentially mild, E-presumably nonpathogenic, and F-nonpathogenic. Mutation resulting in no residual GAA activity in the expressed cells (C% = 0–2) and medium (M% = 0–2), and undetectable GAA protein by western blot (M110 = 1,1; C110 = 1,1; C95 = 1,1; C76 = 1,1), in other words; missing 110 KDa band in medium and missing 110, 95, and 76 KDa bands in cell lysate, is classified as class ‘A’. Class ‘B’ mutation yields normal/subnormal amount of the proper sized GAA protein in the expressed cells but not always in the culture media, said otherwise; missing/visible 110 KDa band in medium and visible 110, 95, and 76 KDa bands in cell lysate (M110 > =1,1/ C110 > =2,4; C95 > =1,1; C76 > =1,1); and along with 0–2% and 0–5% of wild-type activity in medium and cell lysate, respectively. Class ‘C’ mutation usually results in detectable protein at proper sizes (M110 > =1,1; C110 > =2,4; C95 > =2,4; C76 > 2,4), namely visible 110 KDa band in medium and clearly visible 110, 95, and 76 KDa bands in cell lysate; and 0–10% and 2–10% of enzyme activity in the medium and cells, respectively. Class ‘D’ mutation has normal or less amount of standard sized GAA protein (M110 > =2,4; C110 > =3,4; C95 > =3,4; C76 > =3,4), in other words; presence of 110 KDa band in both medium and cell lysate and presence of 95 and 76 KDa bands in cell lysate; and 5–30% of enzyme activity in the medium and cells. For mutations possessing normal or greater amount of protein of proper molecular mass (M110 > =4,4; C110 > =4,4; C95 > =4,4; C76 > =4,4), otherwise speaking; normal/less than normal presence of 110 KDa band in medium and normal presence of 110, 95, and 76 KDa band in cell lysate, those with the residual enzyme measured at 30–60% in medium and cells are assigned to class ‘E’ while those with the residual enzyme > 60% is considered class ‘F’. For more detail information of severity rating, please refer the original publication by Kroos et al. [17].

### Molecular visualization of GAA mutants

The structures of human GAA alone and in complexes with inhibitors [21] were downloaded from the Protein Data Bank (http://www.rcsb.org/pdb/). PyMOL Molecular Graphics System (Schrödinger LLC) was used to display the structural features of GAA protein (PDB: 5NN8) and focusing on the novel mutations identified.

## Results

### Clinical characteristics

Twelve patients including nine males and three females (10 Thai and 2 from other Asian ethnicities) with IOPD were included in the study (Table 2). Eleven patients were the first affected child of the family. Only one patient (patient 11) had a history of parental consanguinity. Excluding the outlier (patient 2), the average age of onset was 4.1 months (range 2–8) and the average age at diagnosis of IOPD was 8.6 months (range 4–22). Patient 2 was noted to have mild tachypnea at 2 weeks, prompting referral to our hospital at 3–4 weeks of age due to a positive history of two previous siblings affected with cardiomyopathy of unclear etiology. The first sib, a 6-month old infant, was documented to have respiratory distress and dilated cardiomyopathy with impaired ejection fraction (EF at 21%), requiring endotracheal tube intubation until death at the age 11 months. The second affected sib had hypertrophic cardiomyopathy with low EF (29%) at the age 4 months and died at 6 months.

**Table 2:** Clinical phenotypes, clinical outcome and family history of 12 IOPD patients

Patient	Age at onset	Age at diagnosis	Manifestation	ECG	ERT/ Clinical outcome	Parental consanguinity
1	7-8 m	9-10 m	hypotonia, delayed motor and cardiomyopathy	short PR interval, BVH	yes, since 11 m./ death around 3y of age	no
2	1-2 m	1-2 m	tachypnea, mild hepatomegaly	short PR interval, BVH, EF 30%	yes, since 4 wk./ favorable outcome at 4 y	no, but two previous deceased children with cardiomyopathy
3	3-4 m	5-6 m	dyspnea	short PR interval, BVH	no, died at 13 m	no
4	3-4 m	7-8 m	dyspnea, hypotonia, delayed motor milestone	short PR interval, BVH	no/ death at 9 m from severe dehydration (diarrhea)	no
5	5-6 m	11-12 m	dyspnea, hypotonia, delayed motor milestone	short PR interval, combined ventricular hypertrophy	death from heart failure and pneumonia at the age of 1.5 y	no
6	5-6 m	21-22 m	dyspnea, poor feeding, FTT, delayed motor milestone	short PR interval, BVH, severe MR	no/ death around 2 y.	no
7	3-4 m	5-6 m	dyspnea, delayed motor milestone, hepatomegaly	short PR interval, BVH, severe MR, SDD, CK 550 u/L, EF 30%	no/ death around 1-2 y.	no
8	1-2 m	3-4 m	tachypnea, hypotonia, mild hepatomegaly	normal PR interval, BVH, SDD, minimal pericardial effusion	no/ death at 13 m.	no
9	3-4 m	5-6 m	hypotonia, delayed motor mille stone, cardiomegaly	short PR interval; BVH, EF 10%, CK 1,010 u/L	no/ death around 1-2 y.	no
10	1-2 m	5-6 m	dyspnea, recurrent aspiration and pneumonia	short PR interval, BVH, SDD	no, died at 10 m.	no
11	3-4 m	7-8 m	hypotonia, hepatomegaly	BVH, SDD	no, died around 1-2 y.	yes
12	3-4 m	5-6 m	hypotonia, pneumonia with respiratory failure and failed endotracheal extubation	short PR, BVH, mild sub-aortic obstruction, SDD, EF 13%, CK 966 u/L	yes, since 6m. / ventilator-dependent at 18 m., motor power grade 3	no

*BVH* Biventricular hypertrophy, *CK* Creatine kinase enzyme, *ECG* Electrocardiography, *EF* Ejection fraction, *ERT* Enzyme replacement

All patients showed classical presentation of IOPD including predominant hypertrophic cardiomyopathy, generalized hypotonia, muscle weakness, and mild hepatomegaly. Short PR interval was not observed in 2 patients (Table 2). Ventricular outflow tract obstruction was noted in one patient, requiring treatment with diuretics and beta-blocker. Almost all patients died within 1–2 years of birth with the exception of those receiving ERT. Clinical outcome especially the age of death was not precise because some patients were referred back to the referring hospitals in rural area, and then contact was lost and estimated age of death was obtained through direct conversation of the referring physicians for those cases. In all but one (patient 8) case, the enzyme activity measured in leukocyte extracts was undetectable or < 1% of normal activity.

Patient 1 received ERT for almost 1 year before the termination of ERT due to deterioration of clinical course and death occurred at 3 years. Patient 2 received ERT since the age of 1 month. Patient 2 was able to sit with support at 8 months, sit unsupported at 12 months and walk with support at 13 months, but could not walk independently until 22 months. The patient said a meaningful word at 14 months and self-fed at 27 months. At the time of this report, patient 2 is 48 months old and has mild myopathic facies; the patient can speak in short sentences and communicate fairly, walk up and down stairs with holding, and attend local preschool. The patient’s weight and height has been around the 25–50 centiles. Patient 2 never experienced significant respiratory/medical problem since the initiation of ERT. CRIM status was not performed before the start of ERT. In addition, glucosidase alpha IgG antibody analysis using enzyme-linked immunosorbent assay (ELISA) at 34 months was negative. Patient 12 received ERT since the age of 6 months, in addition to diuretics and beta-blocker, tube-feeding, and ventilator support. Despite marked improvement of cardiomyopathy and moderate improvement of muscle weakness, patient 12 still has mild macroglossia and is ventilator-dependent at 18-months of age.

### Mutation spectrum

Biallelic mutations of the *GAA* gene were identified in all patients. The GAA activity, genotypes and effect on protein phenotype are shown in Table 3. Seventeen mutations were identified, including 12 missense mutations: c.781G > A (p.Ala261Thr); c.877G > A (p.Gly293Arg), c.1003G > A (p.Gly335Arg), c.1099 T > C (p.Trp367Arg), c.1437G > C (p.Lys479Asn), c.1538A > G (p.Asp513Gly), c.1895 T > G (p.Leu632Arg), c.1935C > A (p.Asp645Glu), c.1933G > C (p.Asp645His), c.1941C > G (p.Cys647Trp), c.1942G > A (p.Gly648Ser) and c.2065G > A (p.Glu689Lys); one nonsense mutation: c.876C > G (p.Tyr292X); one splicing mutation: c.1327-2A > G (leading to exon 9 skipping and resulting in an in-frame deletion of 37 amino acid residues); one in-frame deletion mutation: c.2024_2026delACA (p.Asn675del); and two frame-shift mutations: c.1226insG (p.Asp409GlyfsX95) and c.1411_14delGAGA (p.Glu471fsX5). Among these, the p.Tyr292X, p.Asp513Gly, p.Leu632Arg, and p.Asp409GlyfsX95 variants have not been previously reported.

**Table 3:** GAA enzyme activity and mutations of 12 patients with IOPD

Patient	GAA activity^a^ (% of normal)	Nucleotide changes	Exons	Effects on coding protein	GAA domains	Inheritance	References (ClinVar accession number)
1	0	c.876C>G	5	p.Tyr292X^b^	N-terminal β-sheet	FA	This report (SCV000925966)
		c.1003G>A	6	p.Gly335Arg	N-terminal β-sheet	MO	Kroos et al .[12]
2	0	c.1935C>A	14	p.Asp645Glu	Catalytic GH31	FA	Hermans et al .[13]
		c.1933G>C	14	p.Asp645His	Catalytic GH31	MO	Lin&Shieh [15]
3	0	c.1099T>C	7	p.Trp367Arg	Catalytic GH31	FA	Palmer et al. [16]
		c.1942G>A	14	p.Gly648Ser	Catalytic GH31	MO	Huie et al .[17]
4	0	c.1226insG	8	p.Asp409GlyfsX95	Catalytic GH31	FA	This report (SCV000925969)
		c.2024_2026delACA	14	p.Asn675del	Catalytic GH31	MO	Wan et al. [18]
5	0	c.1538A>G (hom)	10	p.Asp513Gly	Catalytic GH31	MO, FA	This report (SCV000925967)
6	0.43	c.781G>A (hom)	4	p.Ala261Thr	N-terminal β-sheet	MO, FA	ClinVar (SCV000925965)
7	0.05	c.1411_1414delGAGA	9	p.Glu471fsX5	Catalytic GH31	MO	Wan et al. [18]
		c.1933 G>C	14	p.Asp645His	Catalytic GH31	FA	Lin&Shieh [15]
8	1.46	c.877G>A (hom)	5	p.Gly293Arg	N-terminal β-sheet	MO, FA	Hermans et al. [13]
9	NA	c.1941C>G (hom)	14	p.Cys647Trp	Catalytic GH31	MO, FA	Huie et al. [17]
10	NA	c.876C>G (hom)	5	p.Tyr292X	N-terminal β-sheet	MO, FA	This report (SCV000925966)
11	NA	c.1895T>G (hom)	14	p.Leu632Arg^c^	Catalytic GH31	MO, FA	This report (SCV000925968)
12	0.10	c.1327-2A>G	IVS8	Splicing	-	MO	Kroos et al. [11]
		c.1437G>C	9	p.Lys479Asn	Catalytic GH31	FA	Wan et al. [18]

^a^The activity is expressed as a percent relative to normal control (glucose/hr/mg protein); ^b^the mutation creates a *BfaI* restriction site; ^c^the mutant creates *SfcI* restriction site. PCR-restriction digest with respective enzyme revealed no mutation in 50 healthy control. *IOPD* infantile-onset Pompe disease, *NA* not available. Hom is homozygous mutation and FA and MO mean father and mother, respectively

One novel polymorphism c.858 + 24G > C (SCV000925970) were observed in patient 7. Moreover, the variants c.1726G > A (p.Gly576Ser) and c.2065G > A (p.Glu689Lys) known as common pseudodeficiency alleles among Asian populations were presented in patient 2 (heterozygous for p.Gly576Ser) and patient 6 (homozygous for both p.Gly576Ser and p.Glu689Lys) [26] . The p.Tyr292X and p.Leu632Arg mutations create *BfaI* and *SfcI* restriction sites, respectively. These two mutations were not found in 340 control alleles (100 alleles studied by PCR-restriction digest and 240 alleles based on our in-house whole exome database). The p.Ala261Thr and p.Asp513Gly were not present in 240 control alleles in the whole exome database.

**Fig. 1:**
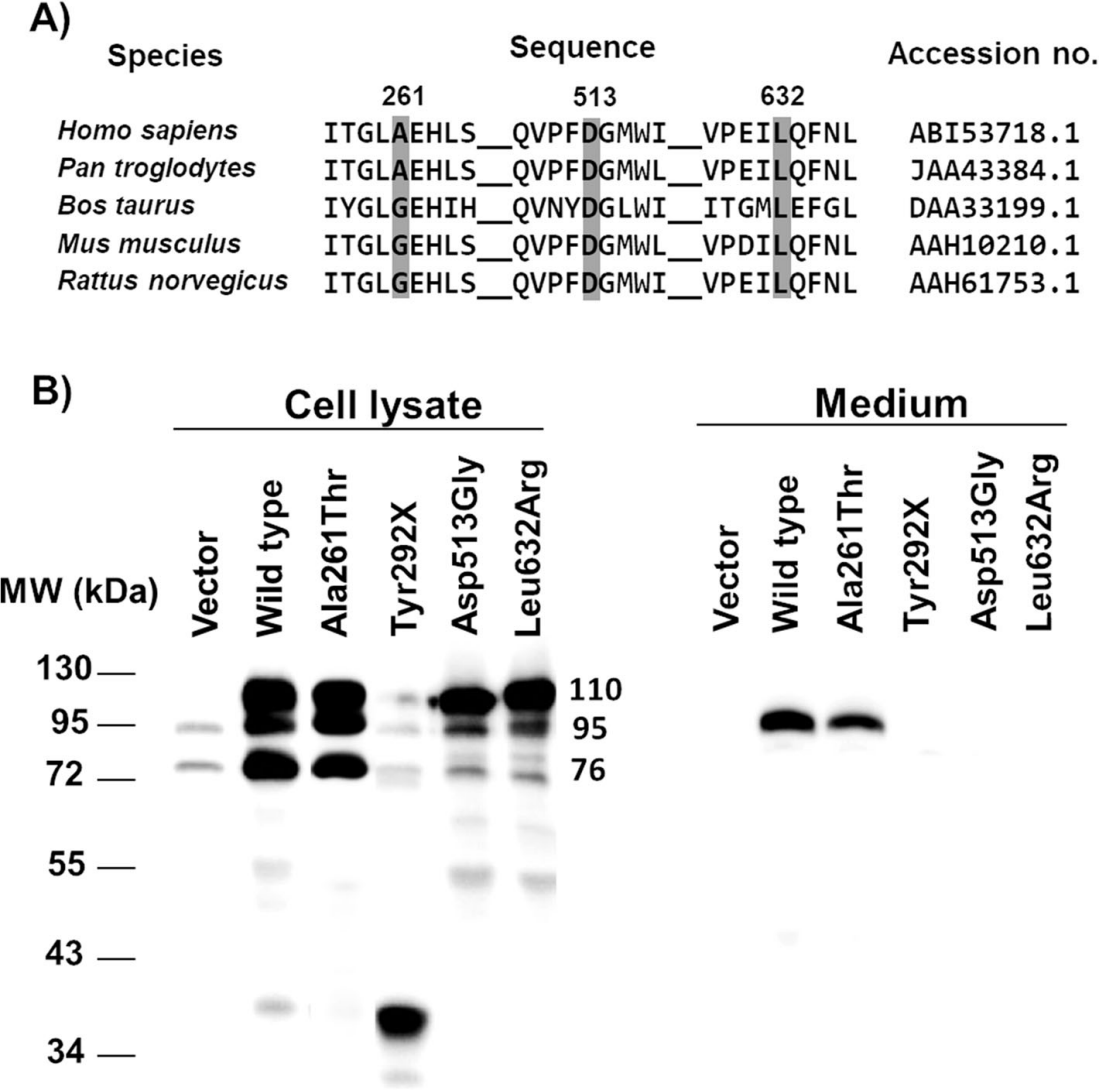
GAA protein analysis. **a** Protein sequence alignment of vertebrate GAA. **b** Western blot analysis of GAA protein in the wild type and mutants

The SIFT, Polyphen-2, PredictSNP2, CADD, DANN, FATHMM, and FunSeq2 online analyses predicted that the three novel missense mutations p.Ala261Thr, p.Asp513Gly and p.Leu632Arg mutations are damaging/deleterious while the GWAVA analysis program indicated neutral change. Comparative in silico analysis of the p.Asp513Gly and p.Leu632Arg showed evolutionary conservation among vertebrate species of Aspartate513 and Leucine632 residues (Fig. 1a). The position of residue 261 is either alanine or glycine (Fig. 1a), but both these amino acids are conserved in having small, non-polar side chains, while that of threonine is significantly larger and more polar.

### Severity class of the mutations studied

Enzyme activity in cells lysates and medium, quantity and quality of the molecular mass of the GAA mutant proteins, upon transient expression in COS-7 cells, and severity class of the four novel mutations identified and the p.Ala261Thr variant are shown in Table 4 and Fig. 1b.

**Table 4:** Specific activity of GAA mutants in transiently transfected COS-7 cells and severity rating

Variant	GAA activity^a^		Severity rating						
	Medium	Cell lysates	M110	C110	C95	C76	M%Wt	C%Wt	Class
Wild type	60.5	631.3 (± 44.5)	4,4	4,4	4,4	4,4	100	100	-
p.Ala261Thr	3.31	478.3	3,4	4,4	4,4	4,4	5.47	75.8	D/E
p.Tyr292X	0	0	1,1	2,4	2,4	2,4	0	0	B
p.Asp513Gly	0	4.8	1,1	3,4	2,4	2,4	0	0.76	B
p.Leu632Arg	0.05	0.1	1,1	3,4	2,4	2,4	0.83	0.02	B

^a^Specific activity of the wild type GAA and its mutants in transiently transfected COS-7 cells (nmol 4-MU/hr/mg protein). M%Wt and C%Wt mean the relative GAA activity normalized by the wild type GAA activity of medium and cell lysates, respectively. M110, C110, C95, C76 are the GAA protein masses detected by western blotting at 100, 95, and 76 kDa from medium (M) and cell lysate (C), respectively. Two digit numbers indicate the quantity and quality of GAA protein; 4 represents as normal while 3, 2 and 1 mean lower quantity and quality of GAA protein, respectively

Compared with wild type GAA, the p.Tyr292X and p.Asp513Gly and p.Leu632Arg mutants displayed enzymatic activities around the level of the negative control from both cell lysate and culture media, whereas the p.Ala261Thr variant exhibited residual activity of approximately 66% in cell extract and 5% in culture medium compared to the wild type control (Table 4). Western blot analysis demonstrated that the p.Ala261Thr mutant exhibited three major bands: 110 kDa precursor (C110), 95 KDa intermediate form (C95) and 76 kDa mature forms (C76), in its intracellular protein pattern, similar to the wild type GAA. As predicted, the p.Tyr292X mutant peptide resulting from early termination of protein synthesis, migrated as a major form at an aberrant molecular mass of 36 kDa (Fig. 1b). The p.Asp513Gly and p.Leu632Arg substitutions appeared to affect the maturation of the 110 kDa precursor to intermediate and mature forms. The 110 kDa precursor was found in culture medium (M) only in p.Ala261Thr but band intensity was obviously less than the wild type GAA, possibly due to an effect on protein transport, while p.Tyr292X, p.Asp513Gly and p.Leu632Arg prohibited secretion of the precursor form completely (Fig. 1b).

The p.Tyr292X, p.Asp513Gly and p.Leu632Arg mutants were assigned to class ‘B’ or potentially less severe mutation. It is unclear whether the p.Ala261Thr mutation should be classified as class D or E since there is much discrepancy between the percentage of normal enzyme activity detected in the cells and medium.

### Molecular structure of the mutant protein

**Fig. 2:**
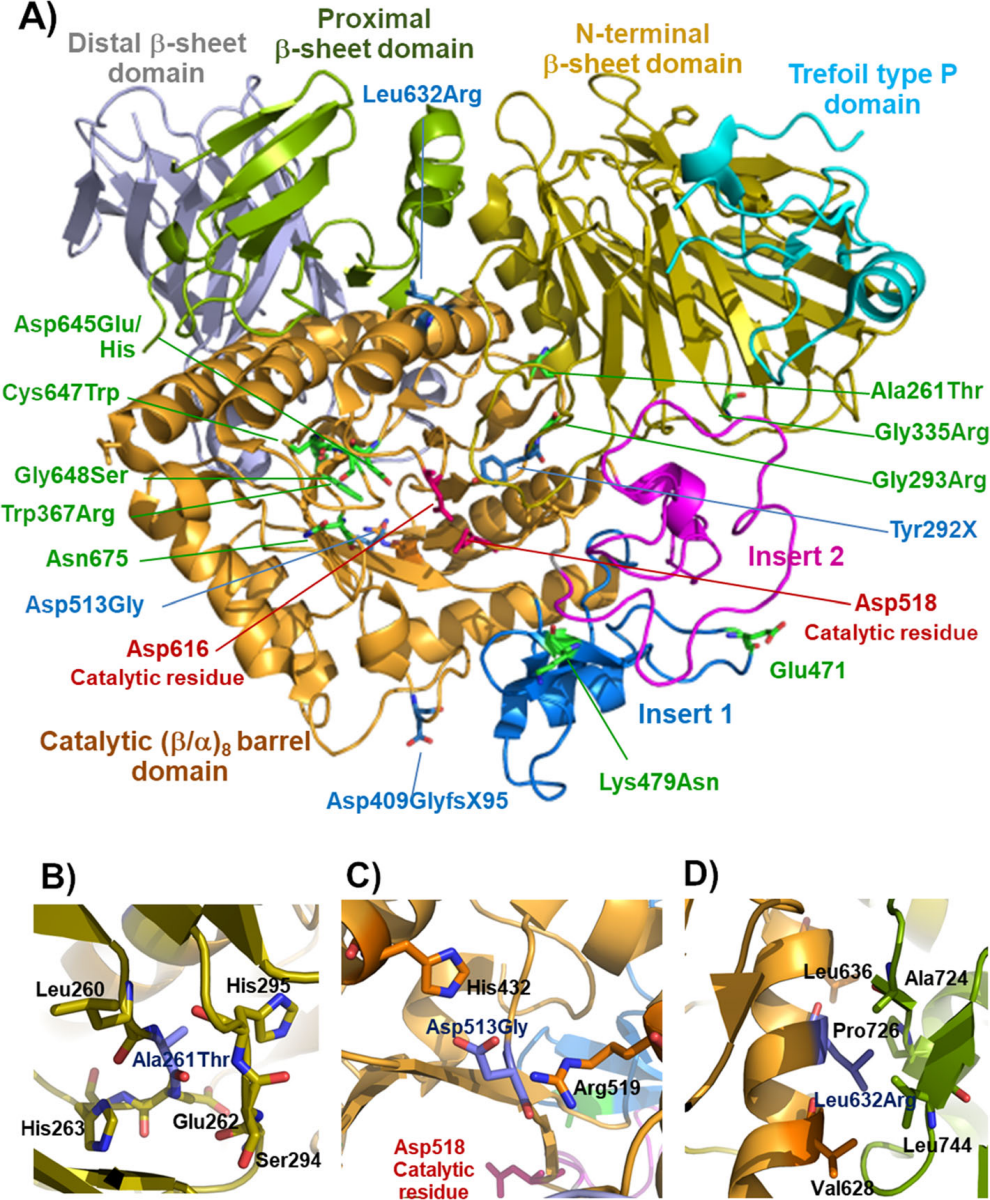
Locations of the mutations in the human GAA structure (PDB: 5NN8, [21]). **a** The positions of the novel mutations are indicated by residue sticks with blue color and the previously reported mutations are indicated by residue sticks with green color, respectively. **b**, **c** and **d** Close-up view of the position p.Ala261Thr, p.Asp513Gly and p.Leu632Arg, respectively

The recent release of structures of human GAA alone and complexed with inhibitors has been useful and allowed better understanding of the structural effects of the GAA mutations [21]. The GAA structure consists of an N-terminal trefoil Type-P domain, followed by a β-sheet domain, the catalytic (β/α)_8_ barrel domain bearing two inserts after β-strands β3 (insert I) and β4 (insert II), and proximal and distal β-sheet domains at the C-terminus. The locations of all identified mutations in this study in the 3D structure are shown in Fig. 2a. The impacts of the novel missense mutations reported in the present study were analyzed based on the 3D structure.

The p.Ala261Thr mutation falls in a loop between the two β-sheets of the N-terminal domain of GAA that interacts tightly with the sixth α-helix of the (β/α)_8_ barrel of the catalytic domain (Fig. 2b). The loop therefore helps to stabilize the interaction between the two domains. In the wild type structure, adding methyl and hydroxyl groups of the threonine and alanine will cause clashes with surrounding residues. Although this may be accommodated by small adjustments in the structure, it is likely to destabilize the interaction between the two domains somewhat. This destabilization may account for this mutant presenting only 5% of wild type GAA in medium of transfected cells, since less of the protein may fold properly and exit out of the endoplasmic reticulum.

The p.Tyr292X truncation, located at N-terminal of β-sheet domain, is predicted to eliminate most of the enzyme, including the catalytic domain, which was confirmed by undetectable GAA activity in both cell lysate and culture medium (Fig. 1b). The p.Gly335Arg mutation was previously rated to have severity of class B [22]. Therefore, it was no surprise that compound heterozygotes between p.Tyr292X and p.Gly335Arg led to IOPD as seen in patient 1 (Tables 2 and 3).

The p.Asp513 residue has been reported to be important for posttranslational modification and intracellular transport of GAA precursor [27]. For p.Asp513Gly, the 110-kDa precursor protein was present in nearly normal amounts, but the 95- and 76 kDa proteins showed great reduction in amount in cell lysate and medium. This data suggests that p.Asp513Gly did not interfere with the synthesis of the precursor protein but disturbed protein processing and secretion (Fig. 1b). The p.Asp513Gly mutant displayed enzyme activity equivalent to negative control in our study, whereas p.Asp513Glu, a previously reported mutation, possessed residual activity around 22% of wild type GAA [27]. The p.Asp513 residue is located at the N-terminus of the fourth β-stand of the catalytic domain, on the other end of which is the catalytic acid/base residue p.Asp518. The interactions of p. Asp513 which help to position the β-strand and thus the catalytic acid/base, include salt bridge hydrogen bonds with p.His432 and p.Arg591 at the C-terminal ends of the second and fourth α-helices of the (β/α)_8_ barrel. Therefore p.Asp513 has a crucial role in stabilizing the (β/α)_8_ structure that is responsible for holding the two catalytic residues, p.Asp518 and p.Asp616, and the substrate in the exact position for hydrolysis (Fig. 2a and c). Replacing the aspartic residue with glycine (p.Asp513Gly) will result in the loss of this charged H-bonding network, which should significantly destabilize this structure.

The p.Asp409 mutation is located in the loop after the second β-strand of the catalytic domain; therefore, the frame shift mutation c.1226insG or p.Asp409GlyfsX95 results in truncated peptide lacking most of the catalytic domain, including the two catalytic residues (p.Asp518 and p.Asp616), hence no activity is expected from the residual protein.

The p.Leu632 residue, mutated in p.Leu632Arg, is situated in helix 6 (the helix between the sixth and seventh beta strands of the (β/α)_8_ structure of the catalytic domain and is packed against the proximal β-sheet domain (Fig. 2a). It is in a hydrophobic environment, surrounded by the side chains of p.Val628, p.Leu636, p.Ala724, p.Pro726 and p.Leu744 (Fig. 2d); therefore, replacing the nonpolar leucine side chain with the highly polar, charged arginine side chain is expected to disrupt the hydrophobic packing of the protein, thereby explaining the lack of enzyme activity.

## Discussion

We described 12 patients with typical presentation of IOPD and fatal outcome early in life, with the exception of three patients who received ERT which modified the natural course of the disease. Patient 1 whose ERT was started when the patient was already on ventilator yielded unsatisfying clinical outcome, ventilator-dependence and complicated pneumonia leading to death. Despite receiving ERT at 4 weeks and having much favorable motor and cardiopulmonary outcomes, patient 2 did not achieve normal milestones. It has been proposed that for better outcome in motor and cognitive function, ERT for IOPD patient should be started before the age of 2 weeks [28, 29]. Without a family history of a previous affected child with Pompe disease, it is quite impossible to make diagnosis and start treatment at such young age. Therefore, it is important that national newborn screening for early diagnosis of IOPD should be implemented which could help the patient achieve full benefit from early treatment with ERT [29]. For patient 12, as expected, the late treatment with ERT (after 1 month) led to an improvement of cardiac manifestation but less effect on respiratory and muscle functions. Moreover, macroglossia in patient 12 may compromise the chance of becoming ventilator-independent.

Hypertrophic cardiomyopathy in the absence of typical short PR-interval and the finding of dilated cardiomyopathy could hinder differential diagnosis of Pompe disease. Most physicians, who are not familiar with Pompe disease, may rely on the presence of short PR along with hypertrophic cardiomyopathy as the diagnostic clue for IOPD, and may not be aware that IOPD may show normal PR interval and dilated cardiomyopathy. We should raise an awareness of these practical points and pitfalls among specialists involved. Despite the fact that the first ERT for Pompe disease became available in 2006, it was not until 2008 that Myozyme was accessible in Thailand, initially by compassionate use and donation from the pharmaceutical company and later commercially available in 2013.

The present study revealed that mutations in the Thai population are more diverse than originally described [30]. Amarinthnukrowh et al. reported the p.Asp645Glu mutation in 8/10 mutant alleles found among Thai patients with IOPD [30]. Including the data from the present study, the prevalence of p.Asp645Glu/His is refined to 32.4% (11/34) and exon 14 was the hot spot for *GAA* mutation at 50% (17/34), followed by exon 5 (Table 3) [30]. Mutation screening of exons 14 and 5, yielding 62% (21/34) detection rate, may serve as a cost-effective strategy for rapid confirmation of IOPD among Thai and Southeast Asian descendants. In addition, The mutation spectrum of this Southeast Asian cohort are partly similar to those described in Chinese and Taiwanese where the p.Asp645Glu is more common among Chinese and Taiwanese IOPD patients, and as a founder mutation [14]. However, our data was markedly different from those reported in Japanese population where the mutation at p.Asp645 was not found in 38 patients and mutations in *GAA*-exons 14 and 5 were rarely identified [24]. It is also important that regional/local mutation database along with CRIM and protein status should be established and publicly accessible so that future patients and treating physicians can benefit from this information.

The severity of all novel missense/nonsense mutations described in the present study are in class B-potentially less severe, implying that the mutants may have different enzyme activity in vivo [17]. It has been observed that most of class B variants were associated with severe infantile onset Pompe disease [17]. Among previously published mutations identified in the present study, p.Gly335Arg and p.Gly648Ser have been listed as class ‘B’ mutations [22]. Despite of yielding near absence of enzyme activity in the in vitro system, p.Gly648Ser was found in an adult-onset Pompe patient when it was found in compound with a mild mutation, c.-32-13 T > G [14, 31].

Previous in vitro expression studies of the mutations p.Gly293Arg, p.Trp367Arg, p.Asp645Glu, and p.Cys647Trp have confirmed significant loss of GAA activity and these variants were found in IOPD patients [23, 31–33]. The p.Asp645Glu variant has been shown to cause delayed intracellular transport, deficient phosphorylation and proteolytic processing [25]. Replacement in the equivalent position p.Asp645Asn and p.Asp645His also result in severe loss of the enzyme activity [23, 31]. The p.Asn675del, p.Glu471fsX5 and p.Lys479Asn variants were previously reported in a Chinese patient with IOPD who carried p.Asp645Glu as the second allele [34]. Up to now, all the frame-shift mutations reported in the database at http://www.pompecenter.nl (updated on May 2016) were ranked as very severe. Though the p.Asp409GlyfsX95 was not tested for functional characterization in the present study, it was convincing that this mutant allele likely falls into severe class ‘A’ mutation due to its nature of frameshift and premature truncation. The p.Asp409GlyfsX95 mutant was found in combination with the p.Asn675del in the patient 4, who exhibited complete loss of GAA activity. The c.1327-2A > G (IVS8) mutation, previously reported in an Arabic patient living in the United Kingdom and listed as a class ‘A’ mutation [17], was found in the last of our patients in association with the p.Lys479Asn mutation.

Mutations found in the IOPD patients of this study, were shown to have undetectable/little enzyme activity with the exception of p.Ala261Thr. The p.Ala261Thr mutation yielded partial reduction of enzyme activity and impaired transport (Fig. 2) and was put into class D/E mutation class by the severity rating system. The results of in silico predictive tools are in agreement in supporting that the residue was potentially damaging. Based on the 3D structure, the position p.Ala261 is located near to the evolutionarily conserved residue p.Glu262 (Fig. 1), with the two residues are in line on the same loop in the N-terminal beta sheet domain. The p.Glu262Lys mutation was previously reported to be a deleterious mutation [35], and it is possible that p.Ala261Thr mutations can be considered to be potentially pathogenic, as found in patient 6, in whom no other deleterious mutation was identified. Notably, patient 6 had classical onset and lethal natural course of IOPD despite of p.Ala261Thr allele being classified as class D/E mutation (Table 2).

Three-fourths (12/16) of the mutations found in this IOPD cohort occurred in the catalytic GH31 domain or resulted in loss of part or all of this domain. The two novel missense mutations, p.Asp513Gly and p.Leu632Arg, were situated in this catalytic domain and were ranked as severe mutations. Homozygous mutation of p.Ala261Thr or p.Gly293Arg which were located in the N-terminal β-sheet domain resulted in little but detectable residual enzyme activity, whereas compound heterozygotes p.Tyr292X/p.Gly335Arg led to complete loss of the GAA activity.

## Conclusion

The broad spectrum of *GAA* mutations described in the present study provide usefulness in confirming the molecular diagnosis of IOPD in the Southeast Asian population of which genetic alterations have been underrepresented in the literature. Almost all the mutations have severity class ‘A’ or ‘B’. The *GAA*-exons 14 and 5 are the hotspots for rapid molecular diagnosis of IOPD especially when enzymatic diagnosis is not a suitable method such as in case of urgent prenatal diagnosis without known familial mutation. These data can benefit rapid molecular diagnosis of IOPD and severity rating of the mutations can serve as a partial substitute for cross reactive immunological material (CRIM) study.

## Data Availability

All relevant data are including in the manuscript. The datasets used and/or analyzed during the current study are available from the corresponding author upon request and on the ClinVar website (https://www.ncbi.nlm.nih.gov/clinvar/) using the ClinVar accession numbers provided in Table 3.

## Abbreviations

CRIM: Cross-reactive immunologic material
EF: Ejection fraction
ERT: Enzyme replacement therapy
GAA: Acid alpha-glucosidase
IOPD: Infantile-onset Pompe disease
LOPD: Late-onset Pompe disease

## References

[1] Kishnani Priya S, Steiner Robert D, Bali Deeksha, Berger Kenneth, Byrne Barry J, Case Laura E, Crowley John F, Downs Steven, Howell R Rodney, Kravitz Richard M, Mackey Joanne, Marsden Deborah, Martins Anna Maria, Millington David S, Nicolino Marc, O’grady Gwen, Patterson Marc C, Rapoport David M, Slonim Alfred, Spencer Carolyn T, Tifft Cynthia J, Watson Michael S (2006). Pompe disease diagnosis and management guideline. Genetics in Medicine.

[2] van den Hout HM, Hop W, van Diggelen OP, Smeitink JA, Smit GP, Poll-The BT (2003). The natural course of infantile Pompe's disease: 20 original cases compared with 133 cases from the literature. Pediatrics.

[3] Leslie N, Bailey L. Pompe disease. GeneReviews® [Internet]. M.P. Adam, H.H. Ardinger, R.A. Pagon, et al., editors.Seattle (WA), University of Washington, Seattle,1993-2018. Initial Posting: August 31, 2007, Last Update: May 11, 2017.

[4] Angelini C, Semplicini C (2011). Enzyme replacement therapy for Pompe disease. Curr Neurol Neurosci Rep.

[5] Hirschhorn R, Scriver CR (2001). Glycogen storage disease type II: acid alpha-glucosidase (acid maltose) deficiency. The Metabolicand Molecular Bases of Inherited Disease.

[6] Hoefsloot LH, Hoogeveen-Westerveld M, Reuser AJ, Oostra BA (1990). Characterization of the human lysosomal alpha-glucosidase gene. Biochem J.

[7] Martiniuk F, Bodkin M, Tzall S, Hirschhorn R (1991). Isolation and partial characterization of the structural gene for human acid alpha glucosidase. DNA Cell Biol.

[8] Moreland RJ, Jin X, Zhang XK, Decker RW, Albee KL, Lee KL (2005). Lysosomal acid alpha-glucosidase consists of four different peptides processed from a single chain precursor. J Biol Chem.

[9] Wisselaar HA, Kroos MA, Hermans MM, van Beeumen J, Reuser AJ (1993). Structural and functional changes of lysosomal acid alpha-glucosidase during intracellular transport and maturation. J Biol Chem.

[10] The Pompe Disease Mutation Database (http://www.pompecenter.nl update on May 2016). Accessed 1 Oct 2018.

[11] Kroos M, Pomponio RJ, van Vliet L, Palmer RE, Phipps M, Van der Helm R (2008). GAA Database Consortium. Update of the Pompe disease mutation database with 107 sequence variants and a format for severity rating. Hum Mutat.

[12] Kroos M, Hoogeveen-Westerveld M, Michelakakis H, Pomponio R, Van der Ploeg A, Halley D (2012). GAA Database Consortium. Update of the pompe disease mutation database with 60 novel GAA sequence variants and additional studies on the functional effect of 34 previously reported variants. Hum Mutat.

[13] Hermans MM, de Graaff E, Kroos MA, Wisselaar HA, Willemsen R, Oostra BA (1993). The conservative substitution Asp-645>Glu in lysosomal alpha-glucosidase affects transport and phosphorylation of the enzyme in an adult patient with glycogen-storage disease type II. Biochem J.

[14] Hermans MM, Kroos MA, van Beeumen J, Oostra BA, Reuser AJ (1991). Human lysosomal alpha-glucosidase. Characterization of the catalytic site. J Biol Chem.

[15] Lin CY, Shieh JJ (1995). Identification of a de Novo point mutation resulting in infantile form of Pompe's disease. Biochem Biophys Res Commun.

[16] Palmer RE, Amartino HM, Niizawa G, Blanco M, Pomponio RJ, Chamoles NA (2007). Pompe disease (glycogen storage disease type II) in Argentineans: clinical manifestations and identification of 9 novel mutations. Neuromuscul Disord.

[17] Huie ML, Tsujino S, Sklower Brooks S, Engel A, Elias E, Bonthron DT (1998). Glycogen storage disease type II: identification of four novel missense mutations (D645N, G648S, R672W, R672Q) and two insertions/deletions in the acid alpha-glucosidase locus of patients of differing phenotype. Biochem Biophys Res Commun.

[18] Wan L, Lee CC, Hsu CM, Hwu WL, Yang CC, Tsai CH (2008). Identification of eight novel mutations of the acid alpha-glucosidase gene causing the infantile or juvenile form of glycogen storage disease type II. J Neurol.

[19] Bali DS, Goldstein JL, Banugaria S, Dai J, Mackey J, Rehder C (2012). Predicting cross-reactive immunological material (CRIM) status in Pompe disease using GAA mutations: Lessons learned from 10 years of clinical laboratory testing experience. Am J Med Genet C: Semin Med Genet.

[20] Zampieri S, Buratti E, Dominissini S, Montalvo AL, Pittis MG, Bembi B (2011). Splicing mutations in glycogen-storage disease type II: evaluation of the full spectrum of mutations and their relation to patients' phenotypes. Eur J Hum Genet.

[21] van der Kraan M, Kroos MA, Joosse M, Bijvoet AG, Verbeet MP, Kleijer WJ (1994). Deletion of exon 18 is a frequent mutation in glycogen storage disease type II. Biochem Biophys Res Commun.

[22] Shieh JJ, Lin CY (1998). Frequent mutation in Chinese patients with infantile type of GSD II in Taiwan: evidence for a founder effect. Hum Mutat.

[23] Hermans MM, van Leenen D, Kroos MA, Beesley CE, Van Der Ploeg AT, Sakuraba H (2004). Twenty-two novel mutations in the lysosomal alpha-glucosidase gene (GAA) underscore the genotype-phenotype correlation in glycogen storage disease type II. Hum Mutat.

[24] Montalvo AL, Bembi B, Donnarumma M, Filocamo M, Parenti G, Rossi M (2006). Mutation profile of the GAA gene in 40 Italian patients with late onset glycogen storage disease type II. Hum Mutat.

[25] Ngiwsara L, Ketudat-Cairns JR, Sawangareetrakul P, Charoenwattanasatien R, Champattanachai V, Kuptanon C (2018). p.X654R IDUA variant among Thai individuals with intermediate mucopolysaccharidosis type I and its residual activity as demonstrated in COS-7 cells. Ann Hum Genet.

[26] Okumiya T, Keulemans JL, Kroos MA, Van der Beek NM, Boer MA, Takeuchi H (2006). A new diagnostic assay for glycogen storage disease type II in mixed leukocytes. Mol Genet Metab.

[27] Koressaar T, Remm M (2007). Enhancements and modifications of primer design program Primer3. Bioinformatics..

[28] Roig-Zamboni V, Cobucci-Ponzano B, Iacono R, Ferrara MC, Germany S, Bourne Y (2017). Structure of human lysosomal acid α-glucosidase-a guide for the treatment of Pompe disease. Nat Commun.

[29] Kroos MA, Mullaart RA, Van Vliet L, Pomponio RJ, Amartino H, Kolodny EH (2008). p.[G576S; E689K]: pathogenic combination or polymorphism in Pompe disease?. Eur J Hum Genet.

[30] Yang CF, Yang CC, Liao HC, Huang LY, Chiang CC, Ho HC (2016). Very Early Treatment for Infantile-Onset Pompe Disease Contributes to Better Outcomes. J Pediatr.

[31] Chien YH, Lee NC, Thurberg BL, Chiang SC, Zhang XK, Keutzer J (2009). Pompe disease in infants: improving the prognosis by newborn screening and early treatment. Pediatrics..

[32] Amarinthnukrowh P, Tongkobpetch S, Kongpatanayothin A, Suphapeetiporn K, Shotelersuk V (2010). p.D645E of acid α-glucosidase is the most common mutation in thai patients with infantile-onset pompe disease. Genet Test Mol Biomarkers.

[33] Fukuhara Y, Fuji N, Yamazaki N, Hirakiyama A, Kamioka T, Seo JH (2017). A molecular analysis of the GAA gene and clinical spectrum in 38 patients with Pompe disease in Japan. Mol Genet Metab Rep.

[34] Huie ML, Chen AS, Brooks SS, Grix A, Hirschhorn R (1994). A de novo 13 nt deletion, a newly identified C647W missense mutation and a deletion of exon 18 in infantile onset glycogen storage disease type II (GSDII). Hum Mol Genet.

[35] Fernandez-Hojas R, Huie ML, Navarro C, Dominguez C, Roig M, Lopez-Coronas D (2002). Identification of six novel mutations in the acid alpha-glucosidase gene in three Spanish patients with infantile onset glycogen storage disease type II (Pompe disease). Neuromuscul Disord.

